# Cure Modelling and Monitoring for Isothermal Processing of Fast-Curing Epoxy Resin

**DOI:** 10.3390/polym18080952

**Published:** 2026-04-14

**Authors:** Patrick Schaible, David Schwaiberger, Sebastian Schabel, Jürgen Fleischer

**Affiliations:** wbk Institute of Production Science, Karlsruhe Institute of Technology (KIT), Kaiserstr. 12, 76131 Karlsruhe, Germany

**Keywords:** thermoset matrix, kinetic model, degree of cure

## Abstract

In liquid composite moulding processes, the curing behaviour of thermoset matrices plays a decisive role in determining manufacturing quality and cycle time. Premature demoulding may lead to insufficiently cured components, whereas excessively long curing times reduce production efficiency. Reliable monitoring and modelling of the curing process are therefore essential for process optimisation. In this study, the cure kinetics of a fast-curing epoxy resin system are modelled using the Grindling kinetic model, which accounts for diffusion-controlled reaction behaviour and vitrification effects. Model parameters are identified using both dynamic and isothermal differential scanning calorimetry (DSC) measurements. In addition, the glass transition temperature is described as a function of the degree of cure using the DiBenedetto relationship. To demonstrate the applicability of the model for process monitoring, an experimental mould equipped with temperature sensors was developed to simulate real-time estimation of the degree of cure during isothermal processing. The predicted degree of cure is validated by post-process DSC analysis of the manufactured samples. Initial comparisons reveal systematic deviations caused by temperature measurement uncertainties. After implementing a temperature correction based on experimentally determined sensor deviations, the predicted degree of cure shows significantly improved agreement with DSC measurements. The results demonstrate that combining kinetic modelling with temperature monitoring enables reliable real-time estimation of the curing state for fast-curing epoxy systems. The study also highlights the critical importance of accurate temperature measurement for curing monitoring and provides insights into the practical implementation of sensor-based monitoring strategies in liquid composite moulding processes.

## 1. Introduction

Thermoset matrix systems such as epoxy resins are widely used in fibre-reinforced composite manufacturing due to their high mechanical performance, chemical resistance, and excellent adhesion properties [1]. Manufacturing processes such as resin transfer moulding (RTM) and other liquid composite moulding techniques increasingly employ fast-curing resin systems to reduce cycle times and improve production efficiency. However, the curing reaction of thermosets strongly influences the final component properties and process stability. In particular, the degree of cure determines the development of mechanical properties, thermal behaviour, and residual stresses during processing. Premature demoulding of a component before sufficient curing has occurred may result in defective or unusable parts, whereas excessive curing times lead to reduced productivity [2,3]. Reliable knowledge of the curing state during processing is therefore essential for optimising process parameters and achieving robust manufacturing conditions. For this purpose, cure modelling combined with process monitoring has become an important tool in modern composite manufacturing. The curing behaviour of thermoset resins is typically described using kinetic models derived from differential scanning calorimetry (DSC) measurements. Well-established models include the Kamal–Malkin model and its extensions, which describe the evolution of the degree of cure based on temperature-dependent reaction kinetics [4]. However, many thermoset systems exhibit diffusion-controlled reaction behaviour when the material approaches vitrification. Under these conditions, the mobility of reactive species decreases significantly, leading to a reduction in the reaction rate. Models that neglect diffusion effects may therefore fail to accurately describe the curing behaviour of fast-curing epoxy systems. To address this limitation, Grindling proposed an extension of the Kamal–Malkin model that incorporates diffusion-controlled reaction kinetics and accounts for vitrification effects through the glass transition temperature. Because the glass transition temperature itself depends strongly on the degree of cure, an additional relationship is required to describe this dependence [5]. The model proposed by DiBenedetto provides a widely used approach for describing the evolution of the glass transition temperature during curing [6]. In addition to kinetic modelling, real-time monitoring of the curing process is of increasing interest for industrial applications. Various techniques have been investigated for monitoring thermoset curing, including dielectric analysis, ultrasonic methods, and temperature-based approaches. Among these methods, temperature monitoring is particularly attractive because temperature sensors are inexpensive, robust, and already widely implemented in industrial processes [7]. When combined with an appropriate kinetic model, temperature measurements can be used to estimate the degree of cure during processing in real time. However, reliable cure monitoring based on temperature data requires accurate modelling of the curing kinetics as well as precise measurement of the resin temperature. Measurement errors or temperature gradients within the mould may lead to significant deviations in the predicted curing state. Consequently, the interaction between kinetic modelling and temperature measurement must be carefully evaluated to assess the practical applicability of such monitoring approaches.

Against this background, the present study investigates the modelling and monitoring of the curing process of a fast-curing epoxy resin system under isothermal processing conditions. Cure kinetics are characterised using both dynamic and isothermal DSC measurements, and the Grindling kinetic model is used to describe the curing reaction while accounting for vitrification effects. The glass transition temperature is modelled as a function of the degree of cure using the DiBenedetto relationship. In addition, an experimental mould equipped with temperature sensors is developed to simulate real-time estimation of the degree of cure during processing. The predicted curing behaviour is validated by DSC measurements of manufactured samples, and the influence of temperature measurement deviations on the prediction accuracy is analysed. The results provide insight into the practical implementation of temperature-based cure monitoring for fast-curing epoxy systems.

## 2. Materials and Methods

### 2.1. Materials

The matrix used is SR8500/SZ8525 epoxy system by Sicomin (Châteauneuf-les-Martigues, France), which is recommended for hot processes and short cycle times for large-scale production [8]. According to the supplier, a curing of 10 min is to be expected at 100 °C. Furthermore, the system exhibits a viscosity of approximately 1000 mPa·s at 25 °C and a density of approximately 1.17 g/cm^3^.

### 2.2. Characterisation

DSC is the most widely applied technique for characterising cure kinetics and is therefore employed in this study [9]. DSC measures the exothermic heat flow released during the cross-linking reaction of a reactive polymer as a function of curing time under both isothermal and non-isothermal conditions. In addition, because a polymer’s heat capacity is temperature-dependent and changes markedly during the glass transition, the glass transition temperature ($T_{g}$) can also be determined. All measurements were performed under a nitrogen atmosphere using a DSC Q200 instrument manufactured by TA Instruments (New Castle, DE, USA).

For the isothermal experiments, the DSC cell was preheated to the target temperature, while the specimens taken from a freshly prepared mixture at room temperature were not preheated. Combined with the small sample masses used, this approach ensures rapid thermal equilibration between the sample and the DSC cell, thereby minimising premature cross-linking. Sample masses of approximately 7–10 mg were analysed. Nevertheless, assuming a zero degree of cure at the start of isothermal measurements conducted at elevated temperatures may be questionable.

Dynamic DSC measurements were carried out at heating rates of 1, 2.5, 5, 10 and 15 °C min^−1^ over a temperature range from −40 °C to 300 °C. Isothermal measurements were performed at 60 °C, 80 °C and 100 °C. The samples were quickly mixed and immediately placed into the preheated DSC cell. After the exothermic reaction had ceased, the samples were rapidly cooled and subsequently reheated to 300 °C at a constant heating rate of 10 °C min^−1^ to determine the residual heat of reaction.

#### 2.2.1. Mathematical Model of Glass Transition Temperature

Although reaction kinetics are typically characterised using both isothermal and non-isothermal DSC experiments, the determination of $T_{g}$ a defined degree of cure is performed here by combining these two temperature programmes. As demonstrated by Fava [10], this approach can serve as an alternative to conventional isothermal measurements.

In this procedure, the specimen is first held isothermally at a specified time–temperature combination. It is then rapidly cooled to a temperature well below the isothermal level (typically below 0 °C) to completely arrest the cross-linking reaction. Subsequently, the sample is reheated to a high temperature at a constant heating rate. Owing to this sequence of isothermal holding, rapid cooling, and reheating, the method is referred to in this study as cyclic DSC.

Reliable $T_{g}$ values can therefore be achieved by starting the heating scan immediately after cooling and ensuring that curing before cooling is conducted at a temperature $T_{c}$ > $T_{g}$, thereby preventing vitrification [11,12].

To model the glass transition temperature in dependence of the degree of cure the approach of DiBenedetto [6] is used in the present work and presented in Equation (1). λ serves as fitting parameter with a value between 0 and 1. $T_{g,0}$ and $T_{g,\infty }$ are the glass transition temperatures in the non-cured state and fully cured state.

(1) $$\frac{T_{g}\left(\alpha \right)-T_{g,0}}{T_{g,\infty }-T_{g,0}}=\frac{\lambda \cdot \alpha }{1-\left(1-\lambda \right)\cdot \alpha }$$

#### 2.2.2. Modelling of Reaction Kinetics

Grindling [5] extended the Kamal–Malkin kinetic model towards considering diffusion-controlled reaction kinetics. Bernath et al. [13] presented in their work an in-depth comparison between the Kamal–Malkin and Grindling model. Therefore, in the present work, only a brief introduction will be presented. Equation (2) presents the key equation of the Grindling model:

(2) $$\frac{d\alpha }{dt}=K_{1}\cdot {\left(1-\alpha \right)}^{n_{1}}+K_{eff}\cdot \alpha^{m}\cdot {\left(1-\alpha \right)}^{n_{2}}$$

The effective reaction rate constant $K_{eff}$ enables the kinetic model to transition between chemically controlled and diffusion-controlled regimes. The parameters $K_{1}$ and $K_{2}$ denote reaction rate constants governed solely by chemical kinetics. They are defined as follows:

(3) $$\frac{1}{K_{eff}}=\frac{1}{K_{2,diff}}+\frac{1}{K_{2}}$$

The reaction rate constant $K_{2,diff}$ describes a diffusion-controlled reaction rate that is governed by the mobility of the reactants. Consequently, it strongly depends on the glass transition temperature $T_{g}$, and on the difference between the current reaction temperature and (*T* − $T_{g}$):

$T>\left(T_{g}+∆T_{g}\right):$

(4) $$K_{2,diff}=K_{2,diff}(T=T_{g}+∆T_{g})\cdot exp\left(-\frac{E_{2,diff}}{R}\cdot \left(\frac{1}{T}-\frac{1}{T_{g}+∆T_{g}}\right)\right)$$

$T_{g}\;\leq T\;\leq \left(T_{g}+∆T_{g}\right):$

(5) $$K_{2,diff}=K_{2,diff}^{T=T_{g}}\cdot exp\left(\frac{c_{1}\cdot \left(T-T_{g}\right)}{c_{2}+T-T_{g}}\right)$$

$T<T_{g}:$

(6) $$K_{2,diff}=K_{2,diff}^{T=T_{g}}\cdot exp\left(-\frac{E_{1,diff}}{R}\cdot \left(\frac{1}{T}-\frac{1}{T_{g}}\right)\right)$$

Equations (4) and (6) follow an Arrhenius-type formulation, incorporating the pre-exponential factors $K_{2,diff}$ and $K_{2,diff}^{T=T_{g}}$ as well as the activation energies $E_{1,diff}$ and $E_{2,diff}$. Equation (5) is based on a modified Williams–Landel–Ferry (WLF) approach and introduces two parameters $c_{1}$ and $c_{2}$ which control the smoothness of the transition between the rubbery and vitrified states:

(7) $$\frac{E_{1,diff}}{R}=T_{g}^{2}\cdot \frac{c_{1}}{c_{2}}$$

(8) $$\frac{E_{2,diff}}{R}=\frac{c_{1}\cdot c_{2}\cdot {\left(T_{g}+∆T_{g}\right)}^{2}}{{\left(c_{2}+∆T_{g}\right)}^{2}}$$

In total, the eleven fitting parameters $A_{1}$, $E_{1}$, $n_{1}$, $A_{2}$, $E_{2}$, $n_{2}$, m, $c_{1}$, $c_{2}$, $K_{2,diff}^{T=T_{g}}$ and $∆T_{g}$ are needed.

## 3. Results and Discussion

### 3.1. Evaluation of DSC Measurements

**Figure 1:**
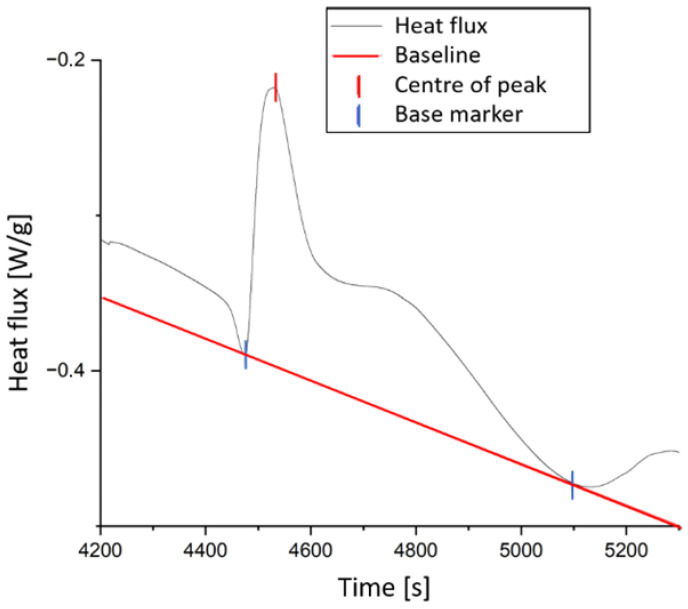
Integration with baseline to determine the residual heat for isothermal DSC measurement at 80 °C.

The degree of cure α depends on the enthalpy released during the DSC measurement and is determined by the formula

(9) $$\alpha =\frac{h(t)}{∆h}=\frac{1}{∆h}\cdot \int_{t_{0}}^{t}q(\hat{t})d\hat{t}$$

where ∆h is the total heat of reaction and q($\hat{t}$) is the heat flow at time t. The total heat of reaction ∆h is calculated by determining a baseline under the exothermic peak and integrating the enclosed area. With the results of the five dynamic DSC measurements, a mean of ∆h is computed. The evaluations are carried out with Origin Professional version 2024b from OriginLab Corporation (Northampton, MA, USA) using the extensions “tangential baseline” and “onset for peaks”. Figure 1 demonstrates the integration with the baseline for the evaluation of the residual heat for an isothermal DSC measurement conducted at 80 °C. Based on this approach, all measurements were evaluated. The results of the isothermal DSC measurements are presented in Table 1, while the results of the dynamic DSC measurements are presented in Table 2. These values provide the basis for the following model fits for glass transition temperature and the Grindling model.

### 3.2. Modelling of Glass Transition Temperature

The glass transition temperatures were determined from isothermal DSC measurements as a function of the degree of cure. Before the curing reaction begins due to the temperature increase, the glass transition can be detected endothermically. Since the glass transition occurs over a range, the glass transition temperature cannot be determined directly. Tangents are applied to determine the initial and final temperatures of the transition range. The mean value is then selected as the glass transition temperature. The results of the glass transition temperatures as a function of the degree of cure are shown in Table 3. Subsequently, the model parameters for the DiBenedetto model are determined using non-linear least-squares in Python v3.12. The results of the model fit are shown in Table 4.

### 3.3. Modelling of the Degree of Cure

The previously created model of the glass transition temperature serves as input for modelling the curing kinetics according to Grindling. The evolutionary algorithm covariance matrix adaptation evolution strategy (CMA-ES) is used for this purpose, which has already proven itself in this regard in the work of Bernath et al. [12]. This is a genetic algorithm with the advantage that not even the order of magnitude of the parameters needs to be known in advance, and a very large solution space can be efficiently investigated. The algorithm works according to the ask-and-tell principle. This means that for each generation, the optimiser requests a number of parameter sets corresponding to the population size. Each parameter set must then be assigned a rating, which in turn is passed on to the optimiser. Based on this, the algorithm adjusts the search field for the next generation. Since the measurements vary significantly in duration and therefore contain different numbers of measurement points, the data set is compressed. This offers the additional advantage of reducing the required computing power or allowing more parameter sets to be tested with the same computing effort.

This only slightly affects accuracy. Two approaches are tested for evaluating the parameter sets. In one run, the curing rate dα/dt is calculated for each measuring point from the curing curves, first from the DSC data and second from the Grindling model. The difference between the values is squared according to the least-squares method. The average value of all error squares is then the quality measure that is passed to the optimiser for each parameter set. In the second run, the absolute values of *α* are compared rather than the curing rates.

**Figure 2:**
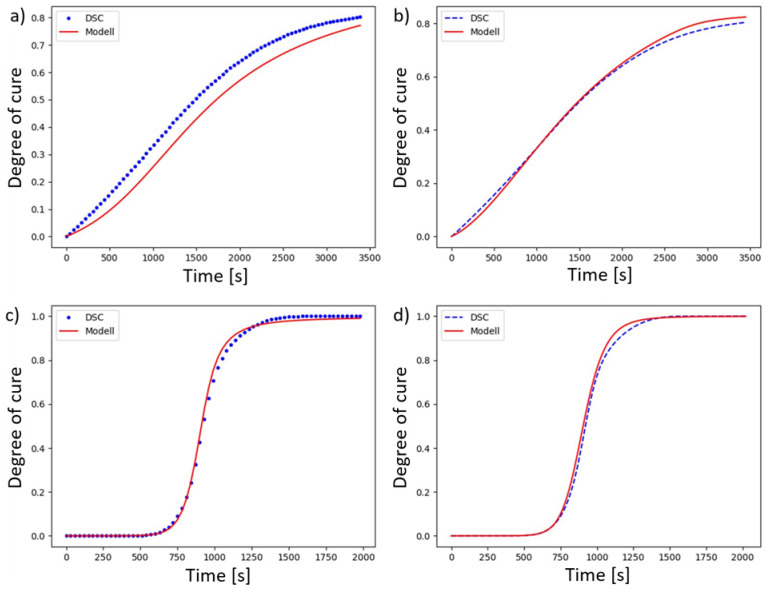
Comparisons of modelling results of (**a**) optimisation with cure rate for isothermal DSC measurement at 60 °C, (**b**) optimisation with absolute degree of cure value for isothermal DSC measurement at 60 °C, (**c**) optimisation with cure rate for dynamical DSC measurement at 10 K/min, and (**d**) optimisation with absolute degree of cure for dynamical DSC measurement at 10 K/min.

For this purpose, the curing curve is calculated using the Grindling model based on the time and temperature data from the DSC curves. The average of the squared difference between $\alpha_{DSC}$ and $\alpha_{Model}$ is used as a measure of quality. Figure 2 shows a comparison of the two approaches for one isothermal and one dynamic measurement each. Although only representative curves are shown for clarity, the comparison between both optimisation approaches was performed using all the available isothermal and dynamic DSC datasets presented in Table 1 and Table 2. A comparison of the isothermal curves shows that optimisation based on the curing rate results in a continuous difference between the DSC and model data, whereas optimisation based on absolute values better fits the measurement curve. Therefore, the latter is chosen for the final modelling. For the final parameter determination, 100 generations with a population size of 1000 were selected. The parameters are shown in Table 5. The average deviation of a point calculated by the model from the DSC value is $\bar{\varepsilon }$ ≈ 0.01854.

### 3.4. Parameter Sensitivity Analysis

The Grindling kinetic model used in this study contains eleven fitting parameters that describe both chemically controlled and diffusion-controlled reaction kinetics. Because a large number of parameters may introduce uncertainty in the model fitting process, a qualitative sensitivity analysis was performed to evaluate the influence of individual parameters on the predicted curing behaviour. The parameters governing the chemically controlled reaction rate, particularly the Arrhenius parameters of the reaction rate constants, have the strongest influence during the early stages of the curing reaction when the material is in the rubbery state. Variations in these parameters primarily affect the initial slope of the curing curve and the time required to reach intermediate degrees of cure. In contrast, the parameters associated with the diffusion-controlled regime mainly influence the later stages of curing when the glass transition temperature approaches the curing temperature. These parameters determine the rate reduction that occurs during vitrification and therefore control the asymptotic behaviour of the curing curve near the final degree of cure. The parameters of the DiBenedetto model, which describe the dependence of the glass transition temperature on the degree of cure, indirectly influence the kinetic model by determining the transition between chemically controlled and diffusion-controlled regimes. Changes in these parameters primarily shift the point at which vitrification occurs during curing. Overall, the analysis indicates that the model predictions are particularly sensitive to the parameters describing the chemically controlled reaction kinetics and to the parameters governing the glass transition behaviour. The diffusion-related parameters mainly affect the final stages of the curing process. These findings highlight the importance of accurate experimental determination of both the kinetic parameters and the glass transition behaviour of the resin system.

### 3.5. Experimental Investigation of the Degree of Cure

**Figure 3:**
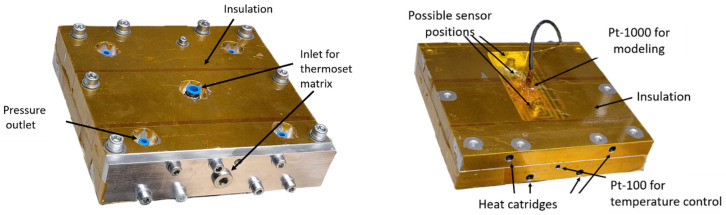
Top and bottom sides of the experimental setup for real-time simulation of the degree of cure.

The test rig uses four 400 W heating cartridges and a JUMO dTRON 316 from JUMO GmbH (Fulda, Germany) compact controller for temperature control. Before closing and screwing the mould, the sealing cord is inserted into the designated groove and a release agent (Frekote 700-NC from Henkel Adhesive Technologies (Düsseldorf, Germany)) is applied. A Raspberry Pi by Raspberry Pi Ltd. (Cambridge, England) is connected to a Pt-1000 sensor from otom Group GmbH (Bräunlingen, Germany) in the mould to gain temperature for the in-line modelling of the degree of cure based on the Grindling model and the previously determined parameters. The mould and the Pt-1000 sensor are covered with polyimide adhesive tape to reduce heat dissipation to the environment. On the right side of Figure 3 the top view of the test rig with the inlet for the thermoset matrix is displayed. On the left side, the bottom view of the temperature sensors is displayed. During the experiments the sensor position in the middle on the direct opposite of the matrix inlet is used. The thermoset matrix can be introduced either in a central position into the mould or from one side via a manual cartridge. The matrix is preheated to the desired temperature and mixed with a static mixer right before entering the mould.

The aim of the tests is to obtain samples with different degrees of cure by combining process time and temperature in as many different ways as possible. It is not possible to plan the tests precisely, as the time required to open the mould and remove the sample is particularly difficult to calculate. It is difficult to achieve low hardness levels, as the material only transitions to a solid state once the reaction has progressed significantly. The used parameter combinations for temperature and duration are provided in Table 6. Furthermore, the estimated degree of cure from the Grindling model is provided for each test. Since the demoulding of #08 and #09 failed due to premature opening of the mould, these two experiments are not considered in the following analysis.

**Figure 4:**
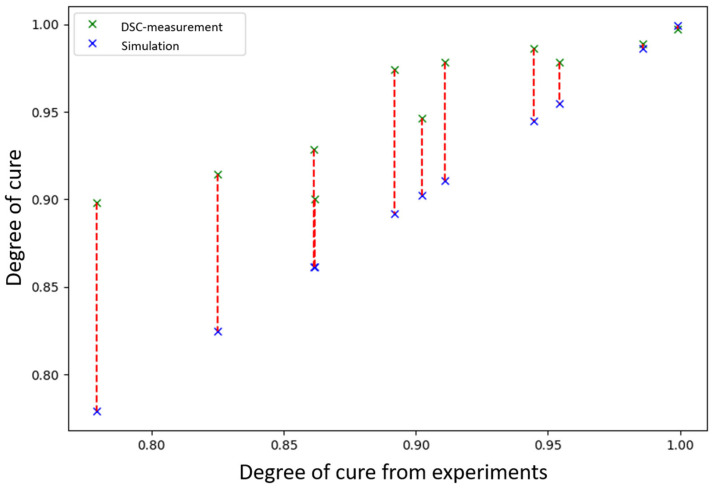
Measured and expected degree of cure with deviation in red from experiments.

The previously produced specimens are now analysed by DSC. For the analysis, a material sample is taken from each test piece. The sampling point is the same for each sample and is located at the level of the Pt-1000 sensor at an identical distance from the heating cartridges. The DSC analysis is carried out using a ‘Q200’ from the manufacturer ‘TA Instruments’ and the evaluation is carried out in the same way as the analyses performed previously. The results of the DSC analysis and the determined values by the model are shown in Figure 4.

Another factor of uncertainty is the temperature. During the experiment, a significant deviation was observed between the Pt-1000 connected to the Raspberry Pi and the Pt-100 of the temperature controller. Since the Pt-100 sensor is located inside the mould, it accurately reflects its temperature. Although the Pt-1000 sensor was covered with insulating foil, a deviation is likely due to the large surface area of the sensor outside the mould. It is assumed that the measurement value of the Pt-100 thermometer is more accurate because it is completely inserted into the mould. Since the temperature controller is not connected to the Raspberry Pi, its temperature measurements were not recorded. The temperature deviations were recorded during the curing tests (see Table 5, Table 6 and Table 7).

The approximated temperature deviation ΔT is determined by the calibration curve approximation:

(10) $$∆T=a+b\cdot T_{Pt-1000|{}^{\circ}C}=-1.6898+0.08025\cdot T_{Pt-1000|{}^{\circ}C}$$

**Figure 5:**
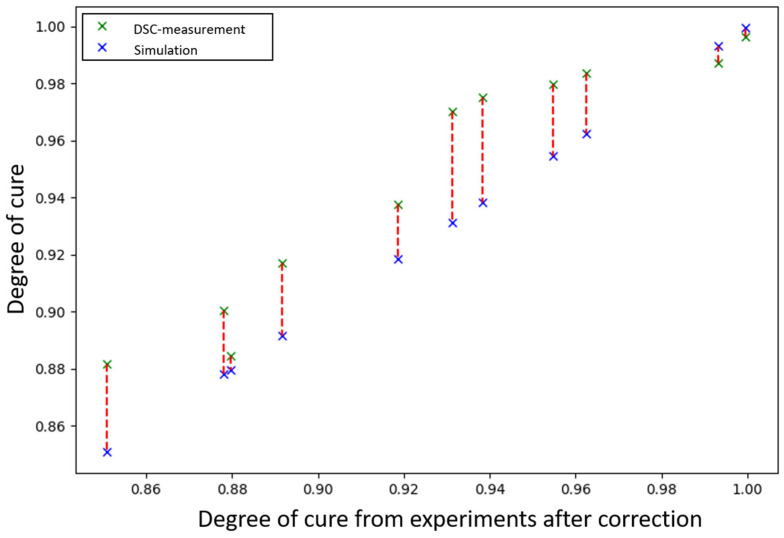
Measured and expected degree of cure with deviation in red from experiments after corrections of temperature values due to deviations between sensors.

The Grindling model is now used again to calculate the curing curve. The temperature data recorded during the tests is used for this purpose, to which the temperature deviation is added according to Equation (10). Particularly in cases where curing has not yet reached a plateau, the temperature difference leads to significantly deviating final degrees of cure. Figure 5 shows the degrees of cure expected by the model at a temperature corrected according to Equation (10) for all test runs, compared to the degrees of cure determined by the DSC, considering the resin-hardener volume ratio. Taking these aspects into account, the deviation between the expected and measured values is significantly lower. The average calculated final degree of cure is only $\Delta \bar{\alpha }$ ≈ 0.021 lower than the value determined by DSC analysis. The maximum deviation determined is $\Delta \alpha_{max}$ = 0.039. The value output by the model therefore remains a conservative estimate. The following aspects may be responsible for this:

- The residual heat in the sample part and the heat released by the exothermic curing reaction cause the reaction to continue after the measurement has ended.
- When the reaction has reached its maximum, a large amount of heat is released. Due to the thermal inertia of the mould and the sensor, this is not recorded immediately. In this case, the resin temperature is higher than the measured value.

Table 7 shows a complete overview of the tests performed. The table presents the degrees of cure determined during the tests, the degrees of cure for higher temperature values calculated using Equation (10), and the degrees of cure measured from the DSC analyses. The test rig developed as part of this work is suitable for producing test pieces from epoxy resin. The Pt-1000 resistance thermometer used has a low measurement deviation of ±0.42 K in the relevant temperature range. Nevertheless, the test evaluation using DSC analysis revealed a significant deviation between the expected and determined degree of curing. Deviations in the temperature measurement are identified as the main reason for this. The Pt-100 sensor of the temperature controller was completely surrounded by the curing mould and measured values that were several degrees higher. It is therefore concluded that positioning the temperature sensor used for curing monitoring on the outside of the mould does not provide reliable measurement data, even if an insulating layer is used. Correcting the determined curing curve based on the measured deviation leads to significantly better results. The predicted final curing degrees are still lower than those indicated by subsequent DSC analyses. Possible reasons for this are the post-curing of the still hot components after removal and the insufficient recording of the temperature increase due to the exothermic curing reaction. The results show that temperature data is suitable for estimating the degree of curing in real time, provided that a sufficiently complex kinetic model with good model parameters is used. However, the experiments carried out have shown that recording the exact temperature of the resin is of utmost importance for reliable predictions. The sensor must be positioned close to the resin and must not be affected by the ambient temperature. The main disadvantage of the method is that it is only a well-founded estimate of the degree of curing and not a measurement of a physical quantity that is directly related to the degree of curing. For reliable measurements, it is also recommended to check the measured values, for example by using a temperature sensor inserted into the resin. With optimised temperature measurement, it is possible to estimate the degree of curing with sufficient accuracy to optimise process times. The cost-effective and straightforward technology makes the process easy to integrate into the workflow. If temperature monitoring of the process is already in place, it can even be implemented by simply upgrading the software. For industrial applications, improved temperature measurement accuracy can be achieved by embedding sensors directly into the mould cavity or by placing them in close proximity to the resin flow path. Such configurations reduce thermal lag and minimise environmental heat losses, thereby improving the reliability of temperature-based cure monitoring.

### 3.6. Uncertainty Analysis of Temperature Measurement

Accurate temperature measurement is essential for the proposed monitoring approach because the degree of cure is estimated from the recorded thermal history using the kinetic model. Therefore, uncertainties in the temperature measurement propagate directly into the predicted curing behaviour. To evaluate the reliability of the monitoring concept, a temperature measurement uncertainty analysis was performed. The uncertainty analysis follows the general approach for measurement uncertainty estimation described in the literature, where individual uncertainty contributions are identified and combined using a root-sum-square formulation. The uncertainty analysis follows the general root-sum-square approach for combining measurement uncertainties, as commonly applied in experimental calibration studies [10]. According to this approach, the combined uncertainty of a measured quantity can be expressed as

(11) $$u_{T}=\sqrt{u_{1}^{2}+u_{2}^{2}+\cdots +u_{n}^{2}}$$

where $u_{i}$ represents the individual uncertainty contributions associated with the measurement process. For the temperature measurement used in the present work, the total uncertainty was assumed to consist of the following main contributions:

- Intrinsic sensor accuracy;
- Uncertainty of the data acquisition system;
- Measurement repeatability;
- Installation-related effects caused by sensor placement and thermal coupling.

The combined standard uncertainty of the measured temperature can therefore be written as

(12) $$u_{T}=\sqrt{u_{sens}^{2}+u_{daq}^{2}+u_{rep}^{2}+u_{inst}^{2}}$$

where $u_{sens}$ denotes the uncertainty associated with the sensor accuracy, $u_{daq}$ the uncertainty introduced by the data acquisition system, $u_{rep}$ the repeatability of the measurement, and $u_{inst}$ the uncertainty caused by installation effects such as sensor position, thermal contact, and environmental heat losses. In the experimental setup used in this study, the Pt-1000 sensor for in-line monitoring was mounted at the outside of the mould and partially exposed to the ambient environment, while the Pt-100 sensor of the temperature controller was fully embedded inside the mould. During the experiments, a systematic temperature deviation between the two sensors was observed. This deviation is mainly attributed to installation-related effects such as heat dissipation from the externally mounted sensor and delayed sensor response during the exothermic curing reaction. To evaluate the influence of temperature measurement uncertainty on the model predictions, the kinetic model was evaluated using perturbed temperature histories. The curing simulation was executed using the modified temperature signals $T\left(t\right)+u_{t}$ and $T\left(t\right)-u_{t}$. The resulting uncertainty of the predicted degree of cure was estimated as

(13) $$u_{\alpha ,T}\approx \;\frac{\alpha \left(T\left(t\right)+u_{T}\right)-\alpha (T\left(t\right)-u_{T})}{2}$$

where $u_{\alpha ,T}$ represents the uncertainty in the predicted degree of cure caused by the temperature measurement uncertainty. To provide a quantitative estimate of the influence of temperature uncertainty on the predicted curing state, the kinetic model was evaluated using a temperature perturbation of ±1 K around the measured temperature history. Such a deviation corresponds to the order of magnitude of the combined temperature uncertainty expected for the sensor system used in this study. For the investigated epoxy system, the numerical evaluation indicates that a temperature deviation of ±1 K typically leads to a variation in the predicted final degree of cure of approximately

(14) ∆α≈±0.015 to 0.025

depending on the curing temperature and the stage of the reaction. Such a deviation corresponds to the order of magnitude of the combined temperature uncertainty expected for the sensor system used in this study. The influence is most pronounced during the main curing phase, where the reaction rate is highly temperature dependent due to the Arrhenius-type behaviour of the kinetic model. In contrast, once the reaction approaches its plateau, the sensitivity of the predicted degree of cure to temperature perturbations decreases. This quantitative estimate confirms that relatively small temperature measurement deviations can lead to noticeable changes in the predicted degree of cure. The result is consistent with the experimental observations, where deviations between the externally mounted Pt-1000 sensor and the embedded Pt-100 sensor resulted in systematic differences between the predicted and experimentally determined curing states. Consequently, accurate sensor placement and thermal coupling are essential for reliable temperature-based cure monitoring. The analysis shows that temperature uncertainty has the largest influence during the main curing phase, where the reaction rate is strongly temperature dependent. Once the curing reaction approaches its plateau, the influence of temperature perturbations becomes smaller. These findings are consistent with the experimental observations, where the externally mounted Pt-1000 sensor initially underestimated the mould temperature and therefore led to a systematic underestimation of the predicted degree of cure. The results highlight that installation-related effects represent the dominant source of uncertainty in the present monitoring setup. For industrial implementation of temperature-based cure monitoring, temperature sensors should therefore be positioned as close as possible to the resin or embedded directly within the mould in order to minimise thermal lag and environmental heat losses.

## 4. Conclusions

In this study, the curing behaviour of a fast-curing epoxy resin system was investigated through a combination of kinetic modelling and experimental validation. Cure kinetics were characterised using dynamic and isothermal DSC measurements and described using the Grindling kinetic model, which accounts for diffusion-controlled reaction behaviour and vitrification effects. The evolution of the glass transition temperature was modelled using the DiBenedetto relationship. To evaluate the applicability of the model for process monitoring, an experimental mould equipped with temperature sensors was developed to simulate real-time estimation of the degree of cure during isothermal processing. The predicted curing behaviour was validated by DSC analysis of samples produced under different curing conditions. The comparison between predicted and experimentally measured degrees of cure showed a generally good correlation. However, systematic deviations were initially observed due to inaccuracies in the temperature measurements obtained from the external temperature sensor. After introducing a temperature correction based on the observed sensor deviation, the agreement between the model predictions and the DSC measurements improved significantly. The corrected predictions remained slightly conservative, which is advantageous for avoiding premature demoulding in practical applications. The results demonstrate that temperature-based monitoring combined with an appropriate kinetic model can provide a reliable estimation of the curing state during the processing of fast-curing epoxy systems. At the same time, the study highlights the critical importance of accurate temperature measurement and sensor placement for achieving reliable predictions.

Future work should focus on improving the measurement of the resin temperature, for example by embedding temperature sensors directly within the resin or by using multiple sensors to capture temperature gradients within the mould. In addition, coupling the kinetic model with heat transfer simulations may allow the influence of exothermic heat generation during curing to be incorporated more accurately. Such developments could further enhance the reliability and industrial applicability of real-time cure monitoring in liquid composite manufacturing processes.

## Figures and Tables

**Table 1:** Evaluation of isothermal DSC analysis.

$\mathrm{Curing}\mathrm{Temperature}\left({}^{\circ}C\right)$	$h_{iso}\left(J\cdot g^{-1}\right)$	$h_{res}\left(J\cdot g^{-1}\right)$	$\alpha_{end}$
60 °C	392.73	95.04	0.8051
80 °C	450.24	40.27	0.9179
100 °C	473.47	18.08	0.9632
120 °C	397.22	9.36	0.9760

**Table 2:** Evaluation of dynamic DSC analysis.

$\mathrm{Heating}\mathrm{Rate}\left(K\cdot {min}^{-1}\right)$	$h_{tot}\;in\left(J\cdot g^{-1}\right)$
1 K/min	445.07
2.5 K/min	462.29
5 K/min	455.78
10 K/min	461.73
15 K/min	448.09

**Table 3:** Glass transition temperature depending on the degree of cure.

Experiment	α	$T_{g}\left(\alpha \right)$
60 °C isothermal	0.8051	66.31 °C
80 °C isothermal	0.9179	101.7 °C
100 °C isothermal	0.9632	109.3 °C
120 °C isothermal	0.9760	116.87 °C
2.5 K/min dynamic	0	−37.2 °C

**Table 4:** Model parameter for glass transition temperature.

Parameter		Unit
$G_{1}$	0.7182	-
$G_{2}$	2.375	-
$T_{g,\infty }$	397.867	K
λ	0.4477	-

**Table 5:** Model parameter for Grindling kinetic model.

Parameter		Unit
$k_{2diff}$	$1.8896\times 10^{-2}$	-
$E_{1}$	$7.7486\times 10^{4}$	$J\cdot {mol}^{-1}$
$E_{2}$	$5.2685\times 10^{4}$	$J\cdot {mol}^{-1}$
$c_{1}$	$4.5655\times 10^{-1}$	-
$c_{2}$	1.4418	-
${\Delta T}_{g}$	278.375	K
$A_{1}$	$1.7574\times 10^{8}$	$s^{-1}$
$A_{2}$	$2.2567\times 10^{5}$	$s^{-1}$
$n_{1}$	3.07641	-
$n_{2}$	1.31032	-
m	$6.25391\times 10^{-1}$	-

**Table 6:** Summary of the experiments for real-time cure simulation.

ID	Duration in s	Average Temperature During Experiment in °C	Degree of Cure Based on Model	Comment
#01	1106.13	97.95	0.9447	
#02	978.32	98.15	0.9346	
#03	686.66	98.05	0.9109	
#04	624.51	97.55	0.8919	
#05	1331.46	78.55	0.8614	
#06	943.13	79.35	0.7793	
#07	1109.28	79.85	0.8249	
#08	-	-	-	Failed due to premature opening of mould
#09	-	-	-	Failed due to premature opening of mould
#10	1016.40	118.15	0.9859	
#11	2427.76	69.65	0.8617	
#12	2320.53	79.65	0.9023	
#13	1983.72	126.15	0.9992	

**Table 7:** Results of experiments after temperature correction and comparison with DSC analysis.

ID	Degree of Cure Based on Model After Correction	Degree of Cure Based on DSC Measurement	Deviation
#01	0.9624	0.9836	0.0212
#02	0.9548	0.9796	0.0248
#03	0.9384	0.9750	0.0366
#04	0.9314	0.9702	0.0388
#05	0.8916	0.9172	0.0256
#06	0.8509	0.8817	0.0308
#07	0.8780	0.9006	0.0226
#10	0.9932	0.9872	0.006
#11	0.8797	0.8844	0.0047
#12	0.9187	0.9377	0.019
#13	0.9996	0.9966	0

## Data Availability

The data presented in this study are available on request from the corresponding author.
